# Improvement in Disability Mediates the Effect of Self-Efficacy on Pain Relief in Chronic Low Back Pain Patients with Exercise Therapy

**DOI:** 10.1155/2022/4203138

**Published:** 2022-08-29

**Authors:** Yuta Shinohara, Kenta Wakaizumi, Aiko Ishikawa, Mari Ito, Reiko Hoshino, Chisato Tanaka, Saki Takaoka, Michiyuki Kawakami, Osahiko Tsuji, Daisuke Fujisawa, Toshiyuki Fujiwara, Tetsuya Tsuji, Hiroshi Morisaki, Shizuko Kosugi

**Affiliations:** ^1^Department of Anesthesiology, Keio University School of Medicine, Tokyo 160-8582, Japan; ^2^Interdisciplinary Pain Center, Keio University Hospital, Tokyo 160-8582, Japan; ^3^Department of Rehabilitation Medicine, Juntendo University Graduate School of Medicine, Tokyo 113-8421, Japan; ^4^Department of Rehabilitation Medicine, Keio University School of Medicine, Tokyo 160-8582, Japan; ^5^Department of Neuropsychiatry, Keio University School of Medicine, Tokyo 160-8582, Japan; ^6^Department of Orthopaedic Surgery, Keio University School of Medicine, Tokyo 160-8582, Japan

## Abstract

**Background:**

The biopsychosocial mechanism by which exercise leads to improvement in chronic low back pain (CLBP) remains unstudied. This prospective cohort study was performed to examine the effectiveness of exercise on pain, disability, and psychological status for CLBP. We also tested path analytic models in which changes in these variables were included.

**Methods:**

CLBP patients who visited the Interdisciplinary Pain Center of Keio University Hospital from July 2018 to April 2020 were included. The propensity score matching was performed between patients who underwent exercise (the exercise group) and those who did not (the control group). At the first visit and at the 3-month follow-up, pain (Numerical Rating Scale (NRS)), disability (Pain Disability Assessment Scale (PDAS)), and psychological status (Pain Self-Efficacy Questionnaire (PSEQ), and Pain Catastrophizing Scale (PCS)) were assessed. Changes in pain and disability at the follow-up were compared between the groups. The relationships between changes in pain, disability, and psychological variables were examined using Pearson's correlation and mediation analysis.

**Results:**

A significantly larger decrease in the PDAS was observed in the exercise group (*N* = 49) than in the control (*N* = 49) (*p* < 0.05). Increased PSEQ scores were significantly correlated with decreased NRS scores in both groups. In the exercise group, decreased PDAS fully mediated the relationship between increased PSEQ and decreased NRS (*P* < 0.05).

**Conclusion:**

Exercise improved disability, and the improved disability by exercise mediated the effect of increased self-efficacy on pain relief in CLBP patients.

## 1. Introduction

Chronic low back pain (CLBP) is associated with psychological distress and functional disability. The fear-avoidance model explains the chronicity of pain as a result of excessive avoidance of physical and social activities based on negative pain beliefs such as fear, catastrophizing, and low self-efficacy [1, 2]. It is a well-established theory that breaking this vicious cycle can lead to improvement in CLBP. In recent years, several guidelines for chronic pain management have highly recommended a multidisciplinary approach, including patient education, behavioral/psychological interventions, and physical therapies [3, 4]. The ultimate aims of these treatments are pain relief and reducing disability, but reducing catastrophizing beliefs and increasing self-efficacy are important processes to achieve these goals [5]. Exercise is the mainstay of multidisciplinary pain management and its effectiveness in reducing pain-related disabilities and negative beliefs has been shown in patients with CLBP [6, 7]. Although improvement in pain and disability is an important outcome of exercise, the biopsychosocial mechanism by which exercise leads to a beneficial outcome is not well studied. One possible mechanism is that exercise improves self-efficacy and disability, thereby relieving CLBP.

The objective of this prospective cohort study was to compare changes in pain and disability at a 3-month follow-up between patients with CLBP who underwent an exercise program and those who did not. We also examined the relationships between changes in pain, disability, self-efficacy, and catastrophizing beliefs in each group and analyzed path models in which changes in these variables were included.

## 2. Methods

### 2.1. Ethics Approval and Consent to Patients

This study was performed in accordance with the Declaration of Helsinki and was approved by the Institutional Ethics Committee of Keio University School of Medicine (authorization number: 2017039). All methods in this study were carried out in accordance with the relevant guidelines and regulations. The ethics committee waived off the requirement to obtain written informed consent since this observational cohort study was noninterventional and therapeutic options were decided according to current clinical practice, independent of participation in the study. However, we provided patients with a written statement regarding the research and obtained verbal consent. In addition, participants were given an opt-out option for the use of data on a tablet device before and after answering questionnaires.

### 2.2. Participants

Patients with CLBP persisting for three months or longer who visited the Interdisciplinary Pain Center of Keio University Hospital from July 2018 to April 2020 were included in this cohort study. Patients with cognitive disorders, cardiopulmonary diseases, bone fractures, cancer, and infections were excluded. A specialized pain management program for CLBP in our interdisciplinary pain center included pharmacotherapy, nerve blocks, and exercise. Treatment options were combined based on the discretion of the pain specialists and the patient's response to treatments. Patients with CLBP were informed about physiotherapy by their pain specialists and referred to rehabilitation physicians if they opted to consult them. Participation in our exercise program was determined by rehabilitation physicians from the pain center; indications, contraindications, and patient background information, such as their accessibility to our center, were all taken into consideration when making these determinations. Patients who underwent exercise therapy were defined in the exercise group and those who did not were defined in the control group.

### 2.3. Evaluation of Pain and Psychological Factors

Patient characteristics, duration of pain, and the site of pain were obtained by medical interview. According to the International Classification of Disease (ICD-11), CLBP was categorized into “primary” and “secondary” CLBP based on neurological examination and imaging findings. CLBP was categorized as “primary” if it was not accounted for by another diagnosis [8, 9]. Pain, psychological factors, and pain-related disability were assessed using self-reported questionnaires. Patients filled out the questionnaires on a tablet device at the first visit and at the 3-month follow-up. The numerical rating scale (NRS) was used to assess the average intensity of pain over the past 24 hours on a scale of 11-point (0 = no pain and 10 = the worst pain imaginable) [10]. The Hospital Anxiety and Depression Scale (HADS) was used to assess anxiety (HADS-A) and depression (HADS-D) [11]. The Pain Catastrophizing Scale (PCS) was used to assess pain-related negative thoughts (i.e., the tendency to view the pain as greater than actual, feel helpless about the pain, and ruminate on the pain). The PCS has ratings from 0 to 52, with each item being rated on a 5-point scale (0: never, 4: always); higher scores indicate a greater degree of pain catastrophizing [12]. The Pain Self-Efficacy Questionnaire (PSEQ) was used to assess the degree of confidence in achieving the desired outcome in daily and social lives despite the presence of pain. The questionnaire consists of 10 items on a self-reported scale from 0 to 3 (0: not at all confident, 6: completely confident). The possible scores in the PSEQ range from 0 to 60, and higher scores indicate a greater degree of confidence to overcome the pain situation [13]. The Pain Disability Assessment Scale (PDAS) was used to assess pain-related disability. The PDAS was developed to measure the daily and social life disabilities of patients with chronic pain. The PDAS consists of 20 items on a self-reporting scale from 0 to 3 (0: pain never interfered with these activities; 3: pain completely interfered with these activities). Five items describe activities using the low back; 7 items describe activities in daily life, and 8 items describe social activities. The possible scores in the PDAS range from 0 to 60, and higher scores indicate a greater degree of disability [14].

### 2.4. Exercise Program

**Figure 1:**
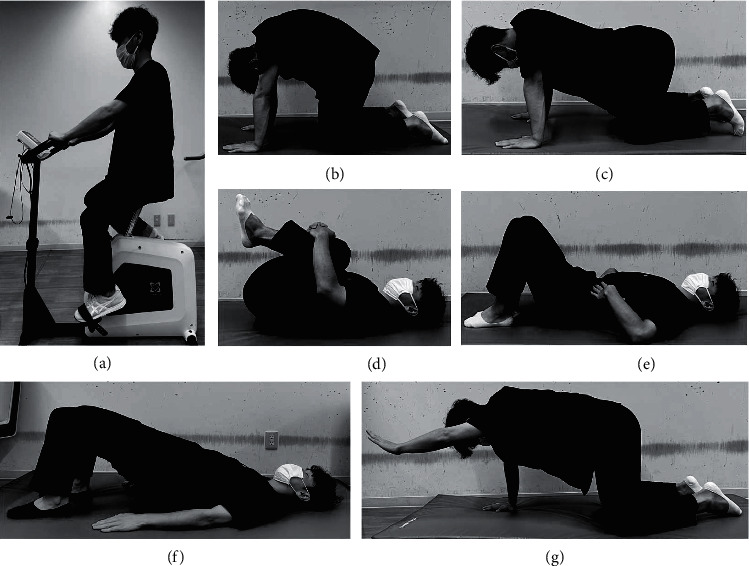
Exercises included in the program. (a) Ergometer: 20–30 minutes. (b)-(c) Cat and dog: 10 repetitions per set. (d) Stretching: 1–3 repetitions per set for each muscle, each held for 30–40 seconds. (e) Draw-in: 10 repetitions per set, each held for 10 seconds. (f) Back bridge: 3 repetitions per set, each held for 10 seconds. (g) Hand and knee. 3 repetitions on both the right and left side per set, each held for 5–30 seconds.

The exercise program was conducted by a physical therapist with 3 years of experience in chronic pain management, and the patient's physical function was rigorously assessed. The program consisted of exercises designed for individual patients, including strength training, stretching, and aerobic exercises (ergometer). To strengthen their core muscles, they performed draw-in, back bridge, and hand and knee exercises. Stretching of the erector spinae muscle, gluteus maximus muscle, hamstrings, and iliopsoas muscles was performed to improve the flexibility of the trunk and lower limbs (Figure 1) [15]. The frequency, intensity, time, and type (FITT) of exercise were tailored according to the individual's conditions, incorporating pacing and gradual progression. In addition to exercise therapy, patient education regarding the mechanisms of chronic pain, self-management techniques, and pacing was provided. The program was conducted on an outpatient basis once or twice a week for 8–12 weeks. Patients were encouraged to perform two sets of each exercise per day and maintain an exercise diary, which was checked by a physical therapist to ensure that they had achieved their individual daily exercise goals. In addition to regular visits to the center, achieving at least 3 days of home exercise in a week was considered compliance with the exercise program.

### 2.5. Outcomes

Changes in the NRS and PDAS at the 3-month follow-up were measured as the two primary outcomes. The secondary outcomes were the changes in the PCS and PSEQ scores at the 3-month follow-up.

### 2.6. Statistical Analysis

Propensity score-matched analyses were conducted to evaluate the effectiveness of the exercise program in improving pain, disability, and pain-related psychological status between the exercise group and the control group. The propensity score was estimated using age, gender, duration of pain, ICD-classification, history of spinal surgery, cotreatment with nerve blocks, and the baseline scores of the NRS, PDAS, HADS, PSEQ, and PCS at the first visit. Patients in the exercise group were matched 1 : 1 with those in the control group by using the nearest-neighbor matching method. Age, body mass index (BMI), and the baseline scores of the NRS, HADS, PSEQ, and PCS were compared between the exercise group and the control group using the unpaired student's *t* test or the Wilcoxon rank sum test for variables that were not normally distributed. Categorical data such as gender, duration of pain (<6 months or ≥6 months), ICD-11 classification (primary pain or secondary pain), and cotreatments such as nerve blocks and pharmacotherapy were compared between groups using the chi-square test for dichotomous variables. After the propensity score matching, changes in the NRS, PDAS, PSEQ, and PCS scores between baseline (at the first visit) and the 3-month follow-up (post-treatment) were examined using the paired *t*-test. Differences over time in pain and disability between the exercise group and control group were examined using repeated measures analysis of variance (ANOVA). A Bonferroni correction for multiple testing was applied (statistical significance; *p* < 0.05/2). The Pearson correlation coefficient was used to examine the relationships between changes in the NRS, PDAS, PSEQ, and PCS scores in each group. For these correlation analyses, Bonferroni correction was applied (statistical significance for each group; *p* < 0.05/5). Next, we investigated the group difference of the correlation coefficient, where Fisher's *z*-transformation was applied to the correlation coefficients and statistical significance was examined under a standard normal distribution. To identify relationships between the correlated variables, we performed a mediation analysis with 5000 permutations using MATLAB 2018a. All statistical analyses other than mediation analyses were performed with JMP ver.14.2.0. Statistical significance was identified by *p* values < 0.05.

## 3. Results

A total of 189 patients with CLBP (54 patients in the exercise group and 135 patients in the control group) were included and followed up for over 3 months. After propensity score matching, 98 CLBP patients (49 patients in the exercise group and 49 in the control group) were analyzed. There were no significant differences in baseline demographics, pain intensity, disability, psychological measures, or the rate of cotreatments during the follow-up period between the exercise group and the control group (Table 1).

Significant improvement was observed in both groups in the NRS, PSEQ, and PCS scores during the follow-up period; the significant improvement in the PDAS scores was observed in the exercise group but not in the control group (Table 2).

### 3.1. The Effectiveness of Exercise Program on Pain and Disability

The repeated measures of ANOVA showed significant differences in the longitudinal data between the exercise group and control group for the PDAS scores, while there were no statistical differences for the NRS between the groups (Table 3). Twenty-four patients in the exercise group visited the center once a week, and the remaining 25 patients visited twice a week. All patients met the criteria for compliance with the exercise program. There were no significant differences in longitudinal data for the PDAS and NRS scores between patients who visited the center once a week and those who visited the center twice a week (PDAS, *F* value = 1.91, *p*=0.17, NRS, *F* value = 0.21, *p*=0.65).

### 3.2. Correlation among Changes in Pain, Disability, and Psychological Variables during the Follow-Up

Table 4 provides the correlation coefficients of changes in scores of the NRS, PDAS, PSEQ, and PCS. In the exercise group, changes in NRS, PDAS, PSEQ, and PCS were all significantly correlated, while only changes in NRS and PSEQ and those in PCS and PDAS were correlated in the control group. A comparison of the correlation coefficients between the groups indicated significantly stronger correlations in the exercise group between changes in the PDAS and NRS and changes in the PDAS and PSEQ scores.

**Figure 2:**
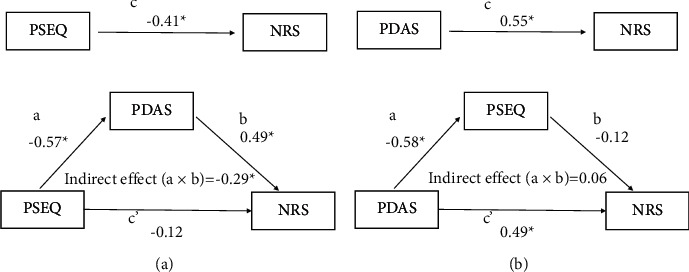
Mediating effect of changes in disability on the relationship between change in self-efficacy and change in pain in the exercise group. (a) The path c indicates the total effect of the whole model. The paths a and b indicate the mediating pathways of change in self-efficacy on change in disability and of change in disability on change in pain. The path c' is the direct effect of change in self-efficacy on change in pain. (b) The paths a and b indicate the mediating pathways of change in disability on change in self-efficacy and of change in self-efficacy on change in pain. The path c' is the direct effect of change in disability on change in pain. PSEQ, Pain Self-Efficacy Questionnaire; PDAS, Pain Disability Scale; NRS, Numerical Rating Scale of pain. ^*∗*^*P* < 0.05.

Based on these correlation analyses, we tested two hypothetical path models in which changes in the NRS, PDAS, and PSEQ in the exercise group were included (Figure 2). The first hypothetical path model showed that a decrease in the PDAS score fully mediated the relationship between increased PSEQ score and decreased NRS score in the exercise group (Figure 2(a)). The second path model indicated that the change in PSEQ had no significant mediating effect on the relationship between the change in the PDAS and NRS scores (Figure 2(b)).

## 4. Discussion

This cohort study revealed significant improvement in pain from the baseline, with or without exercise, in patients with CLBP under specialized pain management. However, a significant improvement in disability was observed only in those who underwent exercise therapy. Improvement in self-efficacy was significantly correlated with improvement in pain in both groups, and the improvement in disability by exercise fully mediated this relationship.

### 4.1. The Effectiveness of Exercise Program on Pain and Disability

The exercise program led to a significant reduction in pain intensity from the baseline but with no superior effect on this goal compared to that of the propensity score-matched controls. This result suggests that a specialized pain management setting with rigorous assessment of multidimensional factors could provide successful pain relief with or without exercise. On the other hand, a significant improvement in disability was observed in the exercise group compared with the control group. Several systematic reviews showed mixed results with regard to the effectiveness of exercise on pain and disability in CLBP, possibly because of heterogeneous interventions [7, 16]. A multicenter randomized controlled trial in Japanese patients with CLBP showed that pain-related disability significantly improved in patients prescribed trunk muscle strengthening and stretching exercises compared with control patients who were treated with nonsteroidal anti-inflammatory drugs [15]. In addition, exercise programs, which include education aiming to correct negative pain behavior, have been reported to improve adherence to rehabilitation [17] and reduce disability and pain in CLBP [18]. Although the exercise program in the current study did not have a standard protocol incorporating a unified FITT, a tailor-made but meticulously supervised exercise plan may have increased adherence to the exercise regimen and resulted in greater benefits in daily activity compared with pharmacotherapy or nerve blocks alone. In this study, 65% of the people in the exercise group were older than 65 years. Although we did not perform an age-stratified analysis, a systematic review showed that in older people, adherence to exercise was influenced by socioeconomic status, health condition, physical ability, cognitive ability, and depression [19]. In older people, a supervised exercise program that considers their functional and psychological state may improve adherence and treatment outcomes. When considering the long-term prognosis of CLBP [20], active therapies such as exercise and education rather than passive ones, such as pharmacotherapy, may aid in self-management of pain through the successful experience of improved disability [21, 22]. Further studies with long-term study periods will clarify whether exercise programs improve the ability of self-management and consequently lead to a reduction in pain in CLBP patients.

### 4.2. Correlation between Changes in Pain, Disability, and Psychological Factors

In the current study, regardless of participation in the exercise program, self-efficacy significantly increased, and this improvement was an important associating factor in pain improvement. In our pain center, healthcare providers repeatedly gave patients advice regarding the importance of self-management during their regular visits. This awareness-building could have led to the observed increase in self-efficacy, which consequently resulted in pain relief. With regard to the relationships between self-efficacy, catastrophizing beliefs, disability, and pain, these variables changed significantly, correlating with each other in the exercise group but were inconsistent in the control group. In addition, our correlation analysis showed that exercise strengthened the relationship between changes in self-efficacy and disability and between the changes in disability and pain intensity. To date, few studies have examined the biopsychosocial mechanism of how exercise could lead to improvement in CLBP [23]. Our results suggest one possible path model in which increased self-efficacy promotes improvement in disability, resulting in pain relief. This potential path model well represents a fear-avoidance model, in which the confidence to confront pain can increase daily activities, thus breaking the vicious cycle and consequently leading to pain relief. A previous study using mediation analysis showed that psychologically informed physiotherapy improved pain-related psychological factors, thereby improving disability in primary care patients with low back pain [24]. This finding could partly support our hypothetical path model, but these mediation analyses did not necessarily imply causal relationships between changes in pain-related variables. Furthermore, interventional studies are required to support the biopsychosocial mechanism we have suggested.

There were several limitations to the interpretation of our data. First, its nonrandomized design with a small sample size limited its ability to control residual confounding. However, we believe that the observer bias and confounding factors could be reduced by using a digital self-rating system on a tablet device and propensity score matching analyses. Second, the study population consisted of patients with relatively severe chronic pain who visited a multidisciplinary pain center and were managed under specialized pain therapy. Therefore, our results may not reflect treatment response in chronic pain patients with less severity. Third, all participants in the exercise group could attend the scheduled sessions. However, since no unified diary was prepared to record their daily exercise, their compliance with home exercise may not have been accurately assessed. Finally, the study subjects did not include all CLBP patients who visited our pain center during the same study period. Although there were no significant differences in demographic, etiological, and psychosocial factors at the first visit between the study subjects and the rest of our patients, our data might have selection bias.

## 5. Conclusions

Exercise therapy improved disability in chronic back pain patients. We suggested a biopsychosocial mechanism in which change in disability by exercise mediates the relationship between improvement in self-efficacy and pain relief. Further studies will be required to support this mechanism and to develop new strategies to provide more beneficial outcomes for exercise therapy.

## Figures and Tables

**Table 1:** Baseline characteristics and cotreatments before and after propensity score matching.

	Whole participants		PS-matched	
	Control (*N* = 135)	Exercise (*N* = 54)	Control (*N* = 49)	Exercise (*N* = 49)
Demographics				
Age (years)	67.5 ± 13.3	64.6 ± 14.1	66.415.3	65.9 ± 14.0
Gender, *n* (%)				
Female	68 (50.4)	31 (57.4)	28 (57.1)	27 (55.1)
Male	67 (49.6)	23 (42.6)	21 (42.9)	22 (44.9)
BMI (kg/m^2^)	23.9 ± 3.2	22.9 ± 4.1^*∗*^	23.7 ± 3.4	22.7 ± 3.8
Duration of pain, *n* (%)				
<6 months	17 (12.6)	5 (9.3)	3 (6.1)	5 (10.2)
≥6 months	118 (87.6)	49 (90.7)	46 (93.9)	44 (89.8)
ICD-classification, *n* (%)				
Primary pain	29 (21.5)	14 (25.9)	11 (22.5)	12 (24.5)
Secondary pain	106 (78.5)	26 (74.1)	38 (77.5)	37 (75.5)
History of spine surgery, *n* (%)	42 (31.1)	18 (33.3)	21 (42.9)	16 (32.7)

Baseline pain and psychological measures				
NRS	5.7 ± 1.8	5.7 ± 1.8	5.7 ± 1.8	5.7 ± 1.9
PDAS	25.1 ± 11.3	28.1 ± 11.1	25.9 ± 10.4	27.5 ± 11.1
HADS-A	6.5 ± 3.9	7.1 ± 4.5	7.0 ± 3.5	6.8 ± 4.4
HADS-D	7.1 ± 4.0	7.6 ± 4.2	7.8 ± 3.7	7.4 ± 4.1
PSEQ	28.3 ± 14.1	24.8 ± 12.0	25.2 ± 12.4	25.9 ± 11.9
PCS	31.4 ± 9.4	33.5 ± 9.4	33.6 ± 8.5	33.3 ± 9.6

Cotreatments during the 3-month follow-up				
Nerve blocks, *n* (%)	119 (88.2)	45 (83.3)	42 (85.7)	42 (85.7)
Pharmacotherapy, *n* (%)	135 (100.0)	54 (100.0)	49 (100.0)	49 (100.0)

Data are expressed as mean ± standard deviation or number (percentage). PS, propensity score; BMI, body mass index; ICD, the International Classification of Diseases; NRS, Numerical Rating Scale of pain; PDAS, Pain Disability Assessment Scale; HADS-A, Hospital Anxiety and Depression Scale-Anxiety; HADS-D, Hospital Anxiety and Depression Scale-Depression; PSEQ, Pain Self-Efficacy Questionnaire; PCS, Pain Catastrophizing Scale.

**Table 2:** Changes in pain, disability, and pain-related psychological status during the 3-month follow-up period.

	Baseline	3 months	*P*
NRS			
Control	5.7 ± 1.8	4.9 ± 2.1	<0.01
Exercise	5.7 ± 1.9	4.3 ± 1.9	<0.0001

PDAS			
Control	25.9 ± 10.4	25.5 ± 11.8	0.74
Exercise	27.5 ± 11.1	22.1 ± 10.4	<0.01

PSEQ			
Control	25.2 ± 12.4	31.3 ± 13.3	<0.01
Exercise	25.9 ± 11.9	32.1 ± 13.4	<0.01

PCS			
Control	33.6 ± 8.5	29.7 ± 11.4	0.02
Exercise	33.3 ± 9.6	28.0 ± 11.9	<0.01

NRS, Numerical Rating Scale of pain; PDAS, Pain Disability Assessment Scale; PSEQ, Pain Self-Efficacy Questionnaire; PCS, Pain Catastrophizing Scale. P, paired *t*-test.

**Table 3:** Comparisons of changes in pain and disability between the control group and the exercise group during a 3-month follow-up.

	*F* value (time)	*P* (time)	*F* value (time^*∗*^exercise)	*P* (time^*∗*^exercise)
NRS				
Baseline	32.8	<0.0001	2.43	0.12
3 months				

PDAS				
Baseline	7.83	0.006	5.87	0.02
3 months				

NRS, Numerical Rating Scale of pain; PDAS, Pain Disability Assessment Scale. P, repeated measures analysis of variance.

**Table 4:** Correlation analyses between changes in pain and disability and psychological status during the follow-up and comparison of the correlations between control vs. exercise.

Control (*N* *=* 49)				
Correlation coefficient (*r*)	ΔNRS	ΔPDAS	ΔPSEQ	ΔPCS
^Δ^NRS	—	0.09	−0.36^*∗*^	0.21
^Δ^PDAS		—	−0.17	0.42^*∗*^

Exercise (*N* *=* 49)				
Correlation coefficient (*r*)	ΔNRS	ΔPDAS	ΔPSEQ	ΔPCS
^Δ^NRS	—	0.47^*∗*^	−0.38^*∗*^	0.41^*∗*^
^Δ^PDAS		—	−0.58^*∗*^	0.62^*∗*^

Comparison of correlation coefficients between control and exercise				
Test statistics *Z*	ΔNRS	ΔPDAS	ΔPSEQ	ΔPCS
^Δ^NRS	—	−2.02^†^	0.10	−1.09
^Δ^PDAS		—	2.38^†^	−1.32

^Δ^Scores at 3 month to scores at baseline; NRS, Numerical Rating Scale of pain; PDAS, Pain Disability Assessment Scale; PSEQ, Pain Self-Efficacy Questionnaire; PCS, Pain Catastrophizing Scale. The Pearson correlation coefficient, ^*∗*^*p* < 0.01. Comparison of correlations between control vs. exercise, ^†^*p* < 0.05.

## Data Availability

The datasets used and/or analyzed during the current study are available from the corresponding author upon request.
